# A Prospective Study of Breast Dynamic Morphological Changes after Dual-plane Augmentation Mammaplasty with 3D Scanning Technique

**DOI:** 10.1371/journal.pone.0093010

**Published:** 2014-03-26

**Authors:** Kai Ji, Jie Luan, Chunjun Liu, Dali Mu, Lanhua Mu, Minqiang Xin, Jingjing Sun, Shilu Yin, Lin Chen

**Affiliations:** Breast Plastic and Reconstructive Surgery Center, Plastic Surgery Hospital, Peking Union Medical College, Chinese Academy of Medical Sciences, Beijing, P. R. China; Sapienza, University of Rome, School of Medicine and Psycology, Italy

## Abstract

**Background:**

The dual-plane technique has been widely used in augmentation mammaplasty procedures. However, there are some concerns about aesthetic contour maintenance for long time after muscle releasing. This study aims to track and analyze breast dynamic morphological changes after dual-plane breast augmentation with three-dimensional (3D) scanning technique.

**Methods:**

Thirteen dual-plane anatomic implant augmentation patients underwent 3D scanning preoperatively (pre-OP) and postoperatively in four time points (1 month: post-1M, 3 months: post-3M, 6 months: post-6M and 12 months: post-12M). The linear distance, breast projection, nipple position, breast volume and breast surface area were measured and analyzed on the 3D models over time.

**Results:**

Compared with post-12M, no significant differences were found in distances of nipple to midline, nipple to inframammary fold and sternal notch to the level of inframammary fold after 6 months in both straight-line distance and its projection on surface. The distances between sternal notch and nipple had no significant difference after post-1M. Breast volume changes had no significant difference after post-3M. The volume and area percentage of upper pole decreased while the lower pole’s increased gradually. The surface showed no significant changes after post-1M. The changes of breast projection had no significance after post-1M either. The nipple moved 1.0±0.6 cm laterally(X axis), 0.6±0.7 cm upward(Y axis) and 2.3±1.1 cm anteriorly (Z axis) at post-12M, and the differences were not significant after post-1M.

**Conclusions:**

3D scanning technique provides an objective and effective way to evaluate breast morphological changes after augmentation mammaplasty over time. Dual-plane augmentation optimizes breast shape especially in the lower pole and maintains stable aesthetic outcome during the 12 months follow-up. Most of the contour changes and the interadaptation with the implant have completed 6 months after operation. Therefore, 6 months could be chosen as a relatively stable observing period in the assessment of postoperative outcomes of dual-plane breast augmentation.

## Introduction

Augmentation mammaplasty has been one of the most commonly performed cosmetic surgical procedures. Subglandular, subpectoral and dual-plane are three commonly used implant pocket locations [1], [2]. Each of these pocket locations has specific indications as well as limitations [2]. Compared with subglandular pocket, pectoral muscle coverage can reduce implant visibility and palpability and decrease risk of capsular contracture. However, it sacrifices lower pole fullness and inframammary fold definition, with increased risk of lateral and superior implant displacement or malposition over time [1], [3]. Dual-plane pocket shapes a more aesthetic lower pole contour and avoids lateral or superior malposition [1], [3]. Dual-plane breast augmentation was introduced with more benefits than other pocket locations, but there are some concerns about the possible increasing risk of palpable or visible implant inferiorly [4]–[6]. To answer this question, evaluation of breast postoperative morphological changes is necessary. In recent papers, three-dimensional (3D) scanning technique has been applied in preoperative evaluation and postoperative outcome assessment for breast surgery [7]–[14]. Its validation and accuracy in breast evaluation have been proved, which provides us an objective tool to analyze breast morphology and symmetry [15]–[20].

However, all of the published studies using 3D scanning technique focused on subpectoral augmentation only, whose results may not apply to dual-plane augmentation [7], [21], [22]. To our best knowledge, there is no published study using 3D scanning technique to evaluate postoperative breast morphological changes after dual-plane augmentation objectively. We analyzed changes in breast contour, projection, volume, surface and nipple position over 12 months follow-up after dual-plane breast augmentation. The study could provide objective and accurate evidences to interpret concerns about dual-plane augmentation and facilitate predictable surgical outcomes.

## Patients and Methods

### Patient Enrollment

Thirteen patients (n = 26 breasts) undergoing primary dual-plane breast augmentation mammaplasty through axillary approach from April 2009 to August 2011 in authors’ department were enrolled into the study. All the patients are Chinese women. Patients with previous breast surgery, congenital breast deformities, significant breast ptosis and other comorbidities were excluded. Allergan 410 anatomic textured silicone implants were used with an average implant size of 238±27.5 ml (range: 205–295 ml).

### Ethics Statement

The study was approved prior to the study by the Research Ethics Committee of the Plastic Surgery Hospital, Peking Union Medical College and Chinese Academy of Medical Sciences. Written informed consent was obtained in accordance with the guidelines of the Research Ethical Committee.

### Three-Dimensional Breast Scanning

The 3D breast images were obtained preoperatively (pre-OP) and postoperatively in four time points (1 month: post-1M, 3 months: post-3M, 6 months: post-6M and 12 months: post-12M) with a noncontact scanner using standardized 3D acquisition protocol, as previously reported [23]. The 3D breast model was analyzed using software (Geomagic Studio 11). The X axis (left/right), Y axis (inferior/superior) and Z axis (posterior/anterior) were determined in coordinate system of the model using our previous protocol [23].

### Surgical Procedure

All procedures were performed using a transaxillary dual-plane technique with endoscopic assistance by the senior author (Jie Luan) [6]. Drains were placed for 3 to 5 days. An elastic band was worn on the upper pole of breast postoperatively to prevent cephalic migration of implants. After surgery, patient satisfaction of their breasts in shape and location was assessed with a scale (from 1, totally dissatisfied, to 10, totally satisfied) at each time point during the 12 months follow-up.

### Linear-Distance Measurements

**Figure 1:**
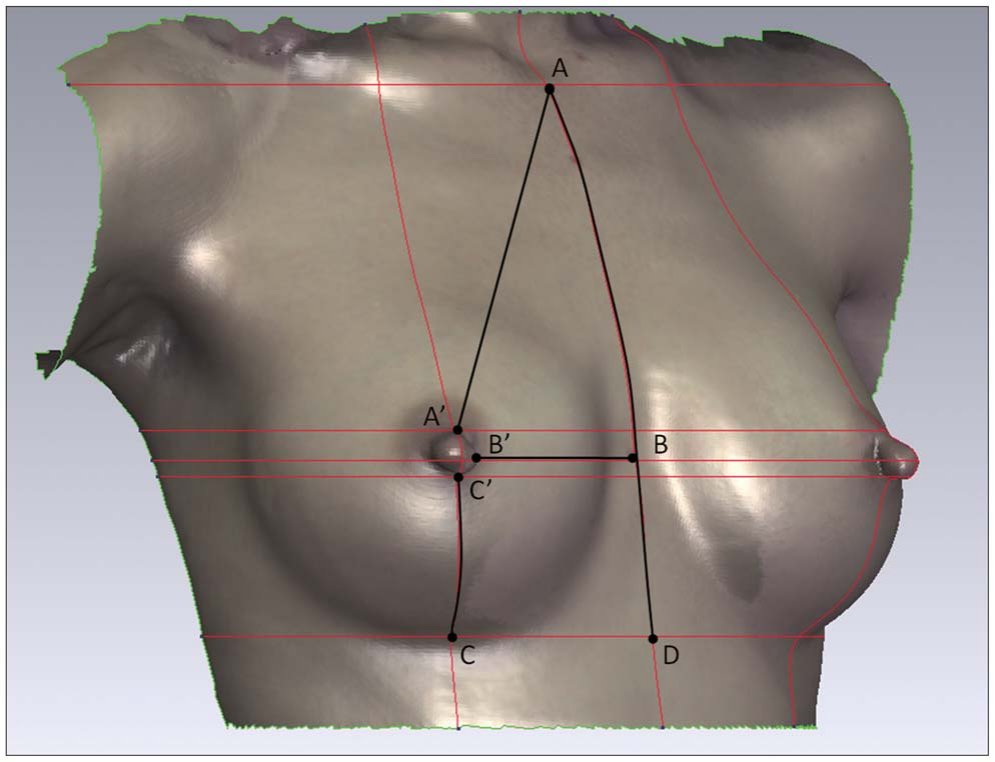
Linear-distance measurements. XZ plane and YZ plane intersected the body surface at each landmarks of body surface to form the red lines. The landmark points for measuring were identified by the intersections of red lines. The horizontal red lines indicate the level of the sternal notch, the upper margin of nipple base, the nipple, the lower margin of nipple base and the inframammary fold. The vertical lines indicate the anterior midline and the middle line of the nipple. Point A = SN (sternal notch), A’ = the superior margin of the nipple base, AA’ = SN-N, B’ = the medial margin of the nipple base, BB’ = N-MD (midline), C’ = the inferior margin of the nipple base, C = the lowest point of the inframammary fold, CC’ = N-IMF (inframammary fold), D = the intersection of the midline and the level of the inframammary fold, AD = SN-LIMF (level of inframammary fold).

**Figure 2:**
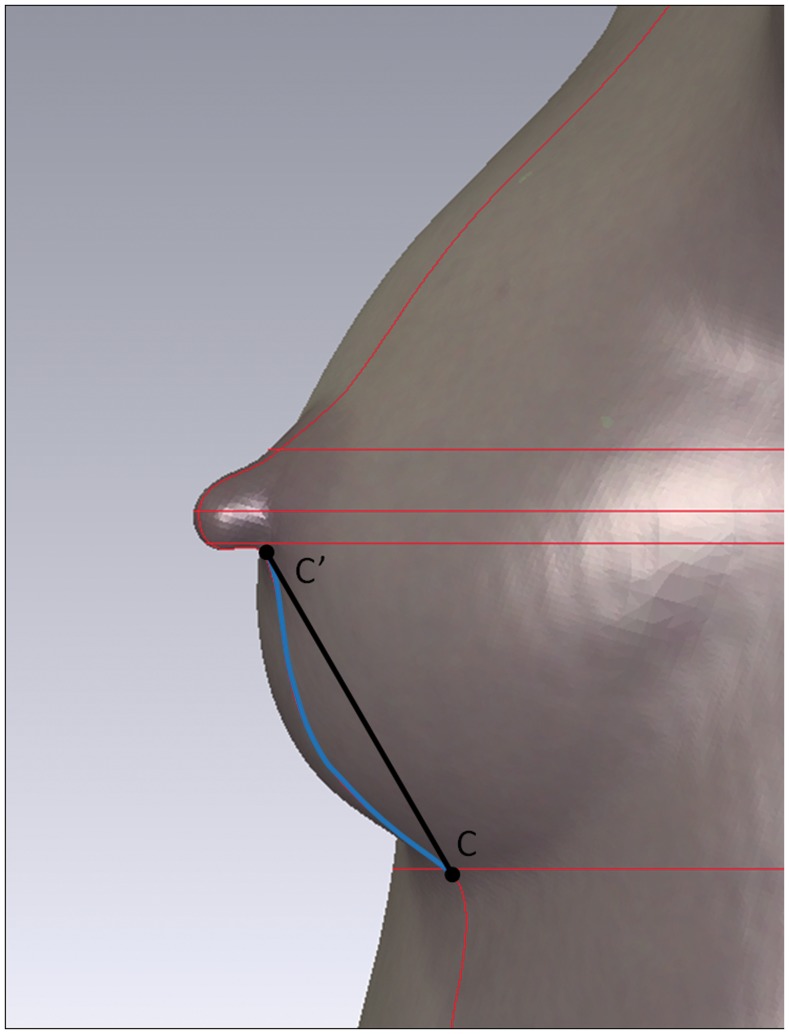
The measurements of straight-line distance and its projection on surface. Point C’ is the lowest point of the nipple base and point C is the lowest point of the inframammary fold. The black line between point C and C’ stands for the straight-line distance of N-IMF. And the blue line between point C and C’ stands for the projection of N-IMF on surface.

Both straight-line linear-distance and its projection on surface between specific anatomical landmarks were measured on the 3D breast model: sternal notch to nipple (SN-N), nipple to inframammary fold (N-IMF), nipple to midline (N-MD) and sternal notch to the level of inframammary fold (SN-LIMF). For good repeatability and accuracy in the measurement, each landmark was identified by the intersection of the horizontal plane (XZ plane) and the sagittal plane (YZ plane) on body surface (Figure 1, Figure 2).

### Breast Projection

**Figure 3:**
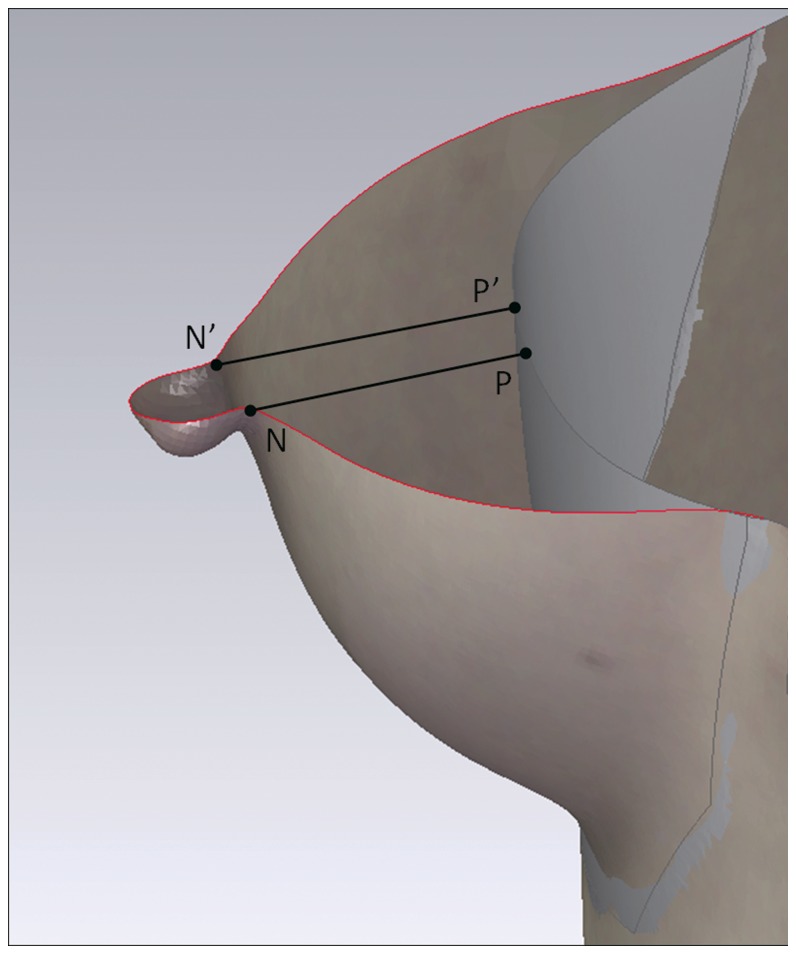
The breast projection and volumetric measurement. This figure shows the horizontal section of the breast in the top view. The gray part is the virtual chest wall simulated by computer to stand for the chest wall. Point N = the medial base of the nipple, N’ = the lateral base of the nipple, NP = the vertical distance from point N to the virtual chest wall and N’P’ = the vertical distance from point N’ to the chest wall. The mean value of NP and N’P’ was calculated as the projection of the breast. The space between the skin surface and the virtual chest wall was measured as the absolute volume of breast.

A virtual chest wall was created based on curvature rate of the peribreast area to stand for the real chest wall [16], [17], [24]. Horizontal sections were obtained through the nipple and the virtual chest wall on each breast to identify the maximal breast projection. The mean value of vertical distance (cm) from the medial and lateral base of the nipple to the chest wall was defined as breast projection (Figure 3).

### Nipple Position

The three coordinates of nipple were measured with sternal notch (SN) as the primary point.

### Breast Volume Measurement and Volumetric Distribution

Total breast absolute volume was measured for each pre- and postoperative 3D model, as previously described [16], [24] (Figure 3). A horizontal plane (XZ plane) was placed through nipple level to divide the marked breast area into upper and lower poles. The volumes of each pole and the volumetric distribution were calculated at each time point.

### Breast Surface Measurement and Areal Distribution

**Figure 4:**
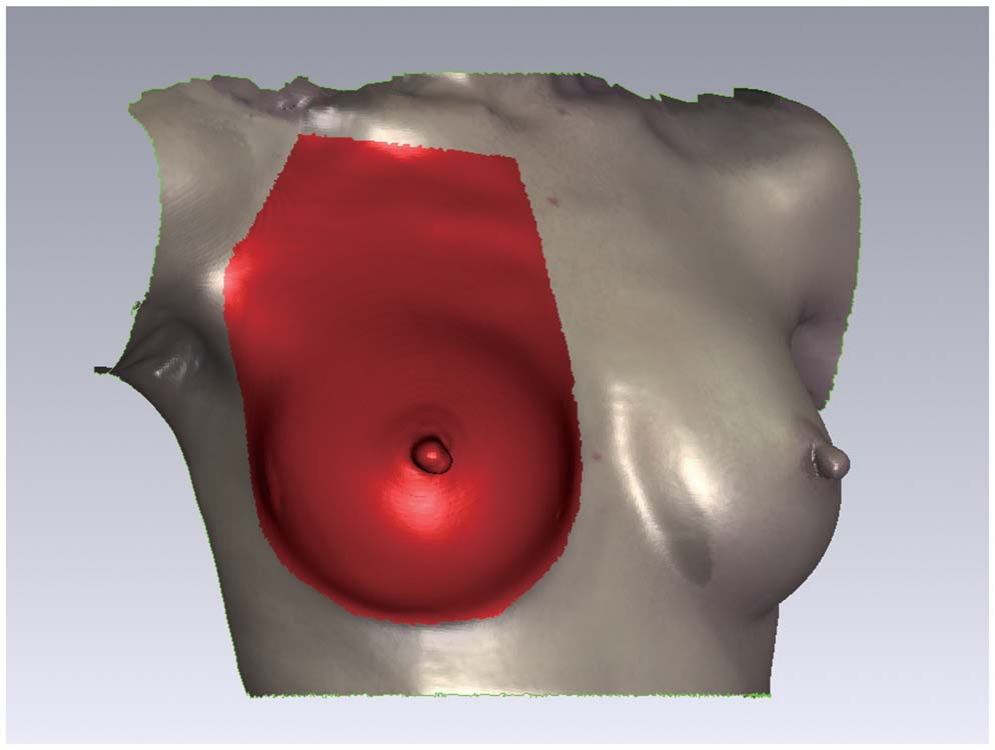
The surface measurement. The red area is the surface area for measurement. The border of the area is marked from 1(IMF) and from lateral offshoot of the breast fold along the front axillary fold and lateral offshoot of the pectoral muscle up to 1 cm below the clavicle. The upper demarcation is 1 cm below and parallel to the clavicle.

The measured breast skin surface area included the area shows in Figure 4. A horizontal plane (XZ plane) was placed through nipple level to divide the marked breast area into upper and lower poles. The total area of the breast surface and the area of each pole were calculated on model at each time point.

### Statistical Analysis

The measurements were conducted at each time point and presented as mean value ± SD. Measurements at post-12M were set as references. One-way ANOVA with repeated measurement was performed to compare between different time points (significance level of p<0.05). The SPSS program version 16 (SPSS, Chicago, IL, USA) was used for analysis.

## Results

### Patient and Implant Characteristics

The mean age of the 13 patients (n = 26 breast) was 28.7±6.3 years (range: 21–42 years), with mean body mass index (BMI) as 18.7±1.3 kg/m^2^ and preoperative breast volume as 41.8±28.1 ml (range: 5–111 ml). The mean value of preoperative difference in chest circumference was 5.6±1.8 cm (range: 2–9 cm). Five out of thirteen patients have had pregnancy before the operation. The patient satisfaction scale had a mean value of 8.8±0.9 at post-12M (range: 7–10) (we defined the score>7 as a satisfied outcome). The change of patients’ satisfaction rate over time was not significant. In all patients, no complications of capsular contracture, bleeding, infection or malposition were observed.

### Breast Linear-Distance Measurements

**Figure 5:**
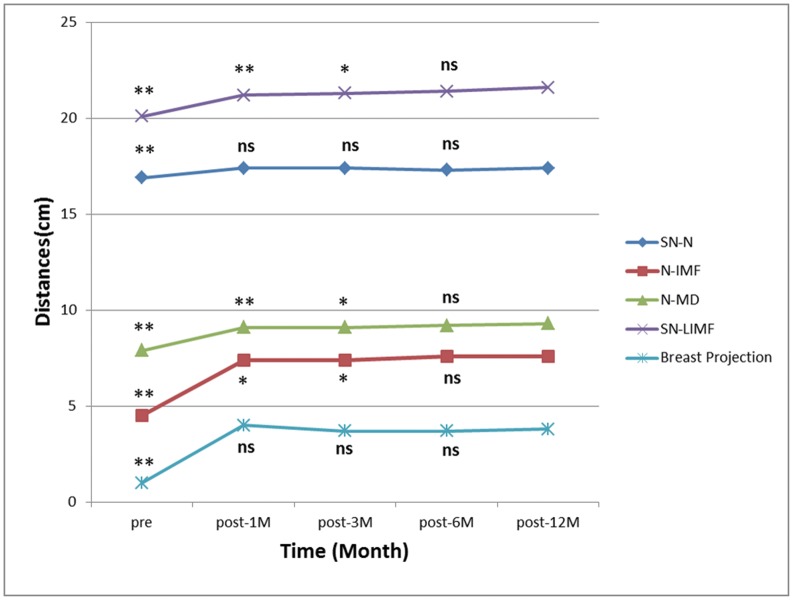
The mean values of linear-distances and the breast projection over 12 months. SN = sternal notch, N = nipple, IMF = inframmary fold, MD = midline, LIMF = level of inframammary fold. The statistical differences at every time point were compared with post-12M in all measurements. ** is p<0.01, * is p<0.05, ns is no significant difference. Post-6M vs. post-12M of N-IMF, N-MD and SN-LIMF distance: p>0.05. Post-1M vs. post-12M of SN-N distance: p>0.05.

All the linear-distance measurements at each time point are summarized in Table 1. The distances of SN-N, N-IMF, N-MD and SN-LIMF significantly increased (p<0.001) after breast augmentation. Both the straight-line distance and its projection on surface of SN-N measurements showed no significant changes after one month (Figure 5). The N-IMF, N-MD and SN-LIMF measurements showed no significant changes in both straight-line distance and projection on surface since post-6M (Figure 5). The SN-N, N-IMF, N-MD and SN-LIMF in linear-distance measurement showed no significant differences between post-6M and post-12M. The IMF dropped 1.1 cm at post-1M and dropped 0.5 cm in the following 11 months.

**Table 1:** Linear-Distance Measurement of Breast.

Parameters	Pre-operative	Post-op	Post-op	Post-op	Post-op
(Mean±SD)		1 month	3 months	6 months	12 months
**Straight-line(cm)**					
** SN-N**	16.6±1.3	17.2±1.3	17.2±1.2	17.1±1.2	17.3±1.1
** N-IMF**	4.4±0.8	7.4±0.7	7.4±1.1	7.6±0.9	7.6±1.0
** N-MD**	7.8±0.4	8.9±0.6	8.9±0.6	9.0±0.5	9.0±0.5
** SN-LIMF**	19.8±1.5	20.9±1.0	21.0±1.0	21.2±0.9	21.4±0.8
**Through-skin(cm)**					
** SN-N**	16.9±1.3	17.4±1.4	17.4±1.3	17.3±1.2	17.4±1.2
** N-IMF**	4.5±0.8	7.4±0.7	7.4±1.1	7.6±0.9	7.6±1.0
** N-MD**	7.9±0.4	9.1±0.6	9.1±0.6	9.2±0.6	9.3±0.6
** SN-LIMF**	20.1±1.5	21.2±1.0	21.3±1.0	21.4±0.9	21.6±0.8

SN, sternal notch; N, nipple; IMF, inframmary fold; MD, midline; LIMF; level of inframammary fold.

### Breast Projection

The average breast projection significantly increased by 3.0 cm after breast implant insertion (pre-OP vs. post-1M, p<0.001) (Table 2) (Figure 5). Thereafter, it decreased slightly, but the difference did not reach a statistical significance (Figure 5).

**Table 2:** Breast Projection and the Projective Distance of SN-N on Axes.

Parameters	Pre-operative	Post-op	Post-op	Post-op	Post-op
(Mean±SD)		1 month	3 months	6 months	12 months
**Projection(cm)**	1.0±0.7	4.0±1.0	3.7±0.7	3.7±0.8	3.8±0.8
**Nipple position (cm)**					
** X axis**	8.4±0.5	9.2±0.5	9.3±0.6	9.4±0.6	9.4±0.6
** Y axis**	14.2±1.2	13.5±1.3	13.5±1.3	13.4±1.1	13.6±1.1
** Z axis**	5.3±1.6	7.5±2.0	7.6±1.5	7.5±1.5	7.6±1.4

SN, sternal notch; N, nipple.

### Nipple Position

**Figure 6:**
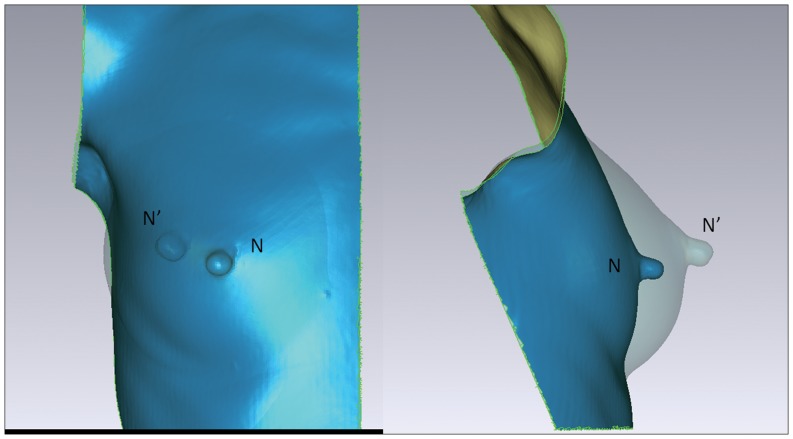
Postoperative nipple shifting. In figures, N = the preoperative nipple position, N’ = the nipple position 12 months after surgery. The left figure is a front view showing the superior and lateral shifting of nipple postoperatively. The right figure is a lateral view showing the superior and anterior shifting postoperatively.

The pre- and postoperative three coordinates of nipple are presented in Table 2. All the changes in three axes were significant (pre-OP vs. post-1M, p<0.001), but there were no significant changes since post-1M (post-1M vs. post-12M, post-3M vs. post-12M and post-6M vs. post-12M, p>0.05). These changes between time point pre-OP and post-12M represented the shifting of nipple in space. That was, the nipple moved 1.0±0.6 cm laterally (X axis), 0.6±0.7 cm superiorly (Y axis) and 2.3±1.1 cm anteriorly (Z axis) after breast implant insertion. From the front view, the nipple moved laterally and superiorly after surgery. From the lateral view the nipple moved superiorly and anteriorly after surgery (Figure 6).

### Breast Volume Measurement and Volumetric Distribution

**Figure 7:**
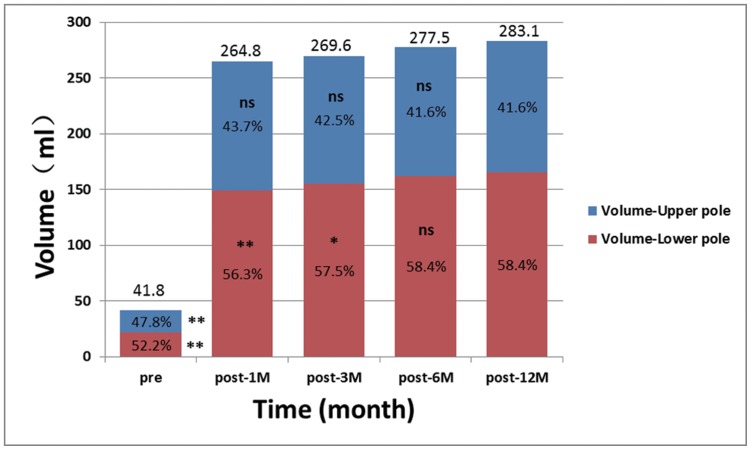
Breast volume and volumetric distribution. A horizontal plane (XZ plane) was placed through nipple level to divide the breast into upper and lower poles. The volumes of each pole and the volumetric distribution were calculated at each time point in percentage. The volumetric percentages of lower pole increased over time while the opposite for the upper pole. The percentage of upper and lower pole at each time point was compared with post-12M. ** is p<0.01, * is p<0.05, ns is no significant difference. Post-6M vs. post-12M of lower pole breast volume: p>0.05. Post-1M vs. post-12M of upper pole breast volume: p>0.05.

The breast volume increased significantly by 241.3 ml after surgery with an average implant size of 238 ml (pre-OP vs. post-12M, p<0.001) (Table 3) (Figure 7). Compared with post-12M, the volumetric changes after post-3M had no significant difference (Figure 7). The volume of breast upper pole increased significantly by 97.7 ml, and the increase was about 143.6 ml in lower pole after surgery (Figure 7). Nearly 60% of the volumetric increase (241.3 ml) derived from the lower pole of breast while 40% from the upper pole. No significant differences of volumetric changes of upper pole were found between post-1M vs. post-12M, post-3M vs. post-12M, post-6M vs. post-12M, respectively (Figure 7). Simultaneously, the volumetric changes of lower pole were significantly different between post-1M vs. post-12M, post-3M vs. post-12M, respectively (Figure 7). However, volumetric changes were not significantly different between post-6M vs. post-12M. The percentage of upper pole surface area decreased gradually while the lower pole’s increased (Table 3) (Figure 7).

**Table 3:** Breast Volume and Surface Measurement.

Parameters	Pre-operative	Post-op	Post-op	Post-op	Post-op
(Mean±SD)		1 month	3 months	6 months	12 months
**Volume (ml)**					
** All**	41.8±28.1	264.8±53.0	269.6±44.7	277.5±62.0	283.1±59.0
** Upper pole**	20.0±15.3	115.6±48.1	114.5±48.9	115.4±57.0	117.7±49.7
** Upper/All**	47.8%	43.7%	42.5%	41.6%	41.6%
** Lower pole**	21.8±14.5	149.2±28.7	155.0±31.9	162.1±30.3	165.4±31.2
** Lower/All**	52.2%	56.3%	57.5%	58.4%	58.4%
**Surface (cm^2^)**					
** All**	334.3±33.3	383.3±38.4	382.4±37.1	382.8±38.3	384.1±38.4
** Upper pole**	234.8±55.0	246.7±58.8	246.3±57.8	243.5±57.2	244.3±56.5
** Upper/All**	70.2%	64.4%	64.4%	63.6%	63.6%
** Lower pole**	99.5±27.6	136.7±31.4	136.1±33.0	139.2±33.5	139.8±31.6
** Lower/All**	29.8%	35.6%	35.6%	36.4%	36.4%

### Breast Surface Measurement and Area Distribution

**Figure 8:**
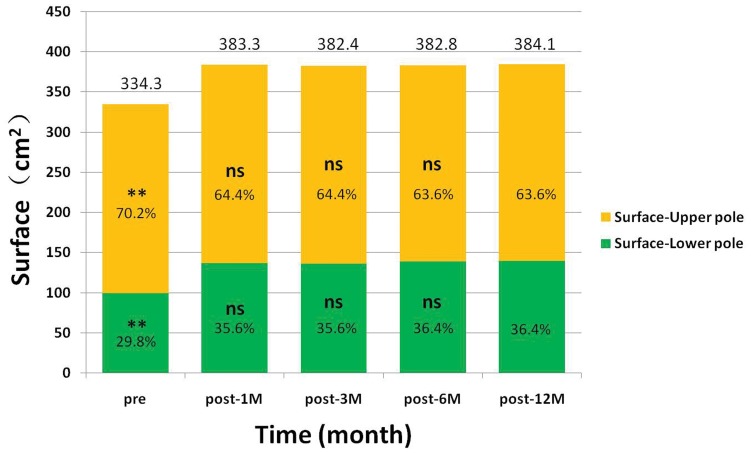
The breast surface and area distribution. A horizontal plane (XZ plane) was placed through nipple level to divide the breast into upper and lower poles. The surface of each pole and the area distribution were calculated at each time point in percentage. The area percentages of lower pole increased over time while the opposite for the upper pole. The percentage of upper and lower pole at each time point was compared with post-12M. ** is p<0.01, * is p<0.05, ns is no significant difference. The upper pole breast surface: post-1M vs. post-12M, post-3M vs. post-12M and post-6M vs. post-12M: p>0.05.

**Figure 9:**
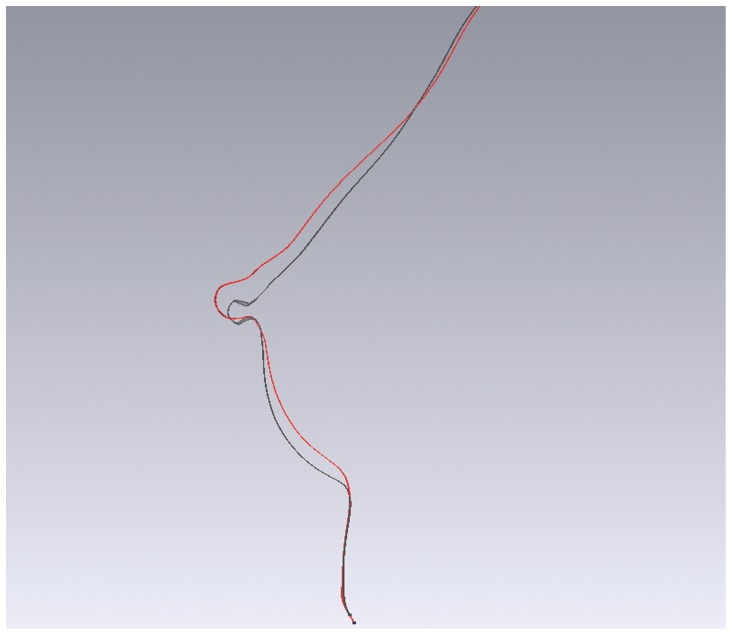
The change of breast contour between post-1M and post-12M in sagittal slice. The red line shows the breast contour of post-1M and the black line shows the post-12M. Post-1M has a relatively plump upper pole versus post-12M and post-12M has a relatively plump lower pole.

The total breast surface increased significantly by 50 cm^2^ after surgery (pre-OP vs. post-12M, p<0.001) (Table 3). No significant differences of area changes were found between post-1M vs. post-12M, post-3M vs. post-12M and post-6M vs. post-12M respectively (Figure 8). The surface area of breast upper pole increased significantly by 10 cm^2^ and the increase was about 40 cm^2^ in lower pole after surgery (Figure 8). Nearly 80% of the surface increase (50 cm^2^) derived from the lower pole while 20% from the upper pole. The percentage of upper pole surface area decreased gradually, while the lower pole’s increased (Table 3) (Figure 8). To illustrate the breast contour changes after surgery, a sagittal section was sliced through the nipple. In paired images of the post-1M and post-12M, we found the breast contour had a relatively full upper pole at post-1M and had a relatively full lower pole at post-12M (Figure 9).

## Discussion

Besides traditional photography, an objective assessment is necessary to describe breast contour changes postoperatively, especially in the lower pole and to confirm the benefits of dual-plane pocket. We use 3D morphological changes in breast to answer the concerns about the possible increasing risk of palpable or visible implant inferiorly of dual-plane pocket [25], [26]. In our study, with dual-plane implant insertion, SN-N distance was relatively stable after post-1M, meanwhile N-IMF distance continuously increased until the relatively stable stage after post-6M. Our data also showed that the volumetric distribution of upper and lower poles changed significantly from the preoperative average of 47.8% in upper pole and 52.2% in the lower to the postoperative average of 41.6% and 58.4% separately (Table 3, Figure 7). Combined with the recorded linear-distance measurement, these findings objectively confirmed that dual-plane technique could preferentially increase fullness in the lower pole of breast. This can also be supported by our results in surface distribution that there was an increased percentage in lower pole and decreased percentage in upper pole after surgery (Figure 8). In addition, proportional volumetric and area percentages between upper and lower poles after surgery demonstrated that dual-plane pocket did not sacrifice the fullness of upper pole when optimize the contour of lower pole. We believe that the releasing of pectoralis muscle increased the fullness of upper pole in a certain extent and this could be the main reason leading to the postoperative redistribution. But the anatomic implant used in our study could be another reason when compared with round implants used in other studies [7], [21]. In our study the nipple had a superior, anterior and lateral shifting after surgery accompanied with a descent of inframammary fold. In our study, the nipple shifting had no significant changes during the 12-months period and the inframmary fold had no significant changes between post-6M and post-12M. Combined with the non-significant decreasing of volumetric distribution percentage in upper pole, there was no superior movement during 12 months follow-up. A convincing evidence of correct implant position should be provided by an imaging system detecting the implant in standing position which is not available now, so 3D scanning technique is the best possibility for now.

In our study, all the linear-distance, volume and surface measurement did not change significantly after 6 months. Moreover eight patients were followed-up over 36 months which showed no obvious differences from post-12M in all the linear-distance, volume and surface measurements.

Eder et al. reported 3D evaluation of breast contour and volume changes following subpectoral augmentation mammaplasty over 6 months with continuous decreasing of N-IMF measurement and increasing of SN-N distance during the postoperative periods [21]. Compared with our study, the different changes in linear distances indicated that dual-plane technique provided more extension on lower-pole. Tepper et al. presented that volumetric distribution of the upper and lower poles of breast did not change with subpectoral augmentation with the average percentage of upper and lower poles about 52.5%±14.7% and 47.5%±14.7% after surgery [7]. However, our data showed different volumetric distribution with the postoperative average of 41.6% in upper pole and 58.4% in the lower (Table 3 and Figure 7). Handel stated that the dual plane releases constriction of pectoralis muscle and facilitates redistribution of the tight skin of the lower pole to accommodate the implant [26]. With reduced pressures from muscle and skin, implants could have more expansion by increasing the arc length from nipple to inframammary fold [2], [26]. Researchers also pointed out that anatomic implant give greater volume support for the lower breast [1], [26]. The sagittal slice in Figure 9 could also support the statements above.

Some researchers presented descent of inframamary fold after breast augmentation as well as drop of the nipple position because of stretching of soft-tissue and the effects of gravity [27], [28]. Tepper et al. found that the nipple height was unchanged after operation [7]. Gryskiewicz indicated the dual-plane technique would dissect the retromammary attachments to the pectoralis major muscle that released the Nipple-Areola Complex (NAC) to rise slightly [5]. Therefore, in our study the nipple showed different shifting comparing with their subpectoral augmentation.

In a recently published paper, Longo et al. presented a predictive formula “BREAST-V” to assess breast volumes based on the anthropomorphic measurements [29]. The predicted weights calculated by the formula had a good linear correlation with the true weights, but the absolute error of the formula was relatively large and the manual measurement error compromised the accuracy of assessment. The 3D scanning technique which could measure breast volumes directly and assess the asymmetry of the chest and breast on the 3D model was superior to the anthropometric measuring in accuracy, precision and reproducibility [23]. But the BREAST-V formula was an easy applied method to predict breast volumes without any equipment. According to their study the formula is more appropriate in sagging breasts, but most of our patients who underwent breast augmentation have relatively small breasts that hardly ptotic. However, the predictive formula of breast volume was very useful in the immediate breast reconstruction for providing reference in selection of implant and autologous tissue. And the predictors included in the formula also gave us an inspiration for our further study.

Our study confirmed that 3D scanning technique was an objective and effective way to evaluate breast morphological changes after dual-plane augmentation mammaplasty over time. Changes in skin and soft-tissue resulting from aging and gravity in long-term could not be ignored although they were hard to predict and measure. We are aware that the small sample size in our study was a limitation to illustrate a representative conclusion. A prospective study of larger volume of cases with subpectoral technique as control group is needed to reach a solid conclusion.

## Conclusion

Three-dimensional scanning technique is an objective and effective way for evaluating breast morphological changes after breast augmentation over time. Based on our study, dual-plane augmentation can optimize breast shape with greater fullness in lower pole. Stable aesthetic outcome can be maintained during 12 months follow-up period. Breasts complete most of the contour changes and the interadaptation with the implant 6 months after endoscopic transaxillary dual-plane augmentation mammaplasty, which indicates 6 months as a minimally required period for postoperative outcome evaluation of dual plane breast augmentation.
